# Ultrasonography and dual-energy computed tomography provide different quantification of urate burden in gout: results from a cross-sectional study

**DOI:** 10.1186/s13075-017-1381-2

**Published:** 2017-07-21

**Authors:** Tristan Pascart, Agathe Grandjean, Laurène Norberciak, Vincent Ducoulombier, Marguerite Motte, Hélène Luraschi, Marie Vandecandelaere, Catherine Godart, Eric Houvenagel, Nasser Namane, Jean-François Budzik

**Affiliations:** 1 Department of Rheumatology, Lille Catholic Hospitals, University of Lille, F-59160 Lomme, France; 2 Department of Medical Research, Biostatistics, Lille Catholic Hospitals, University of Lille, F-59160 Lomme, France; 3 Department of Radiology, Lille Catholic Hospitals, University of Lille, F-59160 Lomme, France; 4 EA 4490, PMOI, Physiopathologie des Maladies Osseuses Inflammatoires, University of Lille, F-59000 Lille, France; 5 Saint-Philibert Hospital, Rue du Grand But, 59160 Lomme, France

**Keywords:** Gout, Ultrasonography, Dual-energy computed tomography, Tophus, Double contour

## Abstract

**Background:**

Ultrasonography (US) and dual-energy computed tomography (DECT) can assess urate burden in gout. The objective of this study was to compare the quantification of urate deposition provided by US to the one provided by DECT.

**Methods:**

Patients with a diagnosis of gout were prospectively recruited to undergo quantification of urate deposition using US and DECT. US examination for tophi and the double contour (DC) sign was performed on the knees and feet and corresponding DECT scans provided volumes of tophi and of overall urate deposition. The primary endpoint was the intra-class correlation coefficient (ICC) of the volume of the index tophus measured by US and DECT and its 95% confidence interval (CI 95%).

**Results:**

Of the 64 patients included, 34 presented with at least one tophus on US. DECT inter-reader agreement for urate deposition was perfect with an ICC of 1 (1–1) and good for the measurement of the index tophus with an ICC of 0.69 (0.47–0.83). The ICC for the measurement of the index tophus between the two techniques was poor with a value of 0.45 (0.1–0.71). The average ratio between the index tophi volume as assessed by DECT and US was 0.65. The number of DC-positive joints did not correlate with DECT volume of overall deposits (Spearman correlation coefficient of 0.23).

**Conclusions:**

DECT measurements of tophi give smaller volumes to the same tophi measured with US, and US signs of urate deposition in joints do not correlate with overall DECT volumes of extra-articular deposition.

**Electronic supplementary material:**

The online version of this article (doi:10.1186/s13075-017-1381-2) contains supplementary material, which is available to authorized users.

## Background

Gout is a common inflammatory form of arthritis that develops after a history of hyperuricemia and subsequent urate deposition within joints and soft tissues [1]. Gout flares occur because of an inflammasome-guided response to the presence of monosodium urate (MSU) crystal deposits [2]. Measuring serum uric acid (SUA) levels provides an instant “snapshot” of the urate overload and is correlated with the risk of flares [3]. Measurement of SUA, however, does not assess the accumulation of urate deposits and therefore cannot estimate how long the road towards urate depletion will be. Morphological examination of the extent of urate deposits can answer this unmet need and is a potential tool for monitoring treatment. International groups of experts have therefore put the development of morphological assessment of urate deposition at the top of the research agenda [4, 5].

Two techniques are particularly promising in that respect: ultrasonography (US) and dual-energy computed tomography (DECT). US performance in the diagnosis of gout is now well-recognised and general consensus has been reached to define which signs to look for and their meaning [6, 7]. Specifically, US allows for the detection of non-clinical tophi and cartilage urate deposition revealed by the double contour (DC) sign [7]. DECT uses two X-ray beams with two different energies, which allows a distinction between chemical entities, and in the case of gout discriminates urate from calcium [8]. DECT provides measurement of the volume of tophaceous and non-tophaceous soft tissue urate deposits with the limitation of spatial resolution [9]. So far, the sensitivity of DECT for urate deposits in soft tissues has been mainly used for diagnostic purposes [10]. Both techniques have independently shown sensitivity to changes in urate deposits visualised after treatment [11–13]. The diagnostic performance of US and DECT has been compared and both exhibit comparable sensitivity for the detection of urate deposition, with potential superiority of DECT [14–16]. Despite the fact that US and DECT are both candidates to quantify urate deposition and monitor urate depletion [17, 18], it is still unknown whether these techniques provide the same quantification of the extent of urate deposition in a given patient.

Here, the aim of the study was to compare the quantification of urate deposition by US and by DECT. In this respect, we investigated the concordance between techniques in assessment of tophus volume assessment, and the correlation between US signs and the volume of urate deposits measured by DECT.

## Methods

### Patients

Consecutive patients with a diagnosis of gout according to American College of Rheumatology (ACR)/European League Against Rheumatism (EULAR) 2015 criteria [19] were prospectively recruited to undergo quantification of urate deposition of the knees and feet using US and DECT. The study was approved by the institutional review board of the Lille Catholic Hospitals and all participants provided informed consent before inclusion in the study.

Demographic details (age, sex, body mass index (BMI), comorbid disorders) and details of the history of gout and medications were recorded, and physical examination (particularly for subcutaneous tophi) was performed at the initial clinical visit. Laboratory testing of SUA and estimated glomerular filtration rate (eGFR) measured by chronic kidney disease (CKD)-EPI or modification of diet in renal disease (MDRD) was to be performed within 2 weeks of the US and DECT examinations.

Patients underwent US and DECT examinations of their knees and feet. US examinations and DECT scans were to be performed with a maximal 2-week interval.

### US examination

Examinations were performed by one of two trained musculoskeletal radiologists (JFB and NN who had 15 and 6 years of experience in radiology, respectively) using an Applio 400 US machine (Toshiba Medical Systems, Tochigi, Japan). High-frequency probes were used: a 12-Mhz probe for the knee examination and an 18-MHz probe the for ankle and foot examination. US examination for tophi was performed in peri-articular structures and soft tissues of the knees, ankles and first metatarsophalangeal joints (MTPs) (tendons, ligaments, Hoffa pad, skin and subcutaneous tissues) [7]. US examination for the DC sign was performed on the femoro-patellar joints, talo-crural joints and first MTPs [7]. The volumes of the tophi were determined and the largest US tophus was selected as the index tophus. Tophus volume was measured using its three maximal dimensions with subsequent automated calculation by the US software.

### Computed tomography (CT) data acquisition and image reconstruction

All scans were performed using a single-source CT device (Somatom Definition Edge; Siemens, Erlangen, Germany). The patients were positioned feet first in a supine position. The knees and feet were scanned axially in two separate acquisitions performed consecutively on the same day. All scans were performed with the same image protocol, acquisition at 128 × 0.6 mm and pitch of 0.7. For each body part, two scans were acquired with tube potentials of 80 kV and 140 kV. Depending on the scanned body region, quality reference tube currents ranged between 62 and 260 mAs. Automated attenuation-based tube current modulation was used in all examinations.

Axial images with soft (B30f) and bone (B70f) convolution kernels were reconstructed with a 1-mm slice thickness and an increment of 1 mm. Post-processing of DECT was performed by the radiologists using dedicated software (syngo.via VB10B, syngo Dual Energy Gout; Siemens), following parameters described elsewhere [20]: UH threshold 150; iodine ratio 1.4; material definition ratio 1.25; resolution 4; air distance 5; and bone distance 10. Two kinds of images were reconstructed for each body part. First, volume-rendered 3D images in which urate crystal deposits were coded in green were reconstructed with a bone tissue convolution kernel (B70f). These images allowed straightforward overview of MSU deposits. Second, multi-planar reformations associating images reconstructed with a soft tissue kernel (B30f) and coloured images were reconstructed. The aspect of the final fusion images could be changed by modulating the relative percentages of the morphological and coloured images from 0 to 100% with a slider.

### CT data analysis

Two musculoskeletal radiologists (JFB and NN) independently evaluated the multi-planar reformations and volume-rendered 3D images. Apart from the localisation of the index tophus given to them by the study coordinator (TP), both radiologists were blinded to the US results.

For each body part, the total volume of urate crystal deposits was automatically quantified in cubic centimetres by the software. Both readers were aware of the known artefacts of DECT for urate deposit detection [21]. When such artefacts were present, a cropping tool was used to suppress them. In a second step, the index tophus was identified on volume-rendered 3D images. A cropping tool was used to isolate the tophus and measure its inner volume. For each index tophus, multi-planar reconstructed fusion images were analysed to confirm the location. These images were also helpful in challenging cases, to better identify the tophus boundaries and to avoid tophus overestimation. If no tophus was visible, the DECT volume of the index tophus was defined as 0.

### Statistical analysis

Statistical analysis was performed using the R software (version 3.2.5). Quantitative variables are expressed as mean and standard deviation, and qualitative variables as number and percentage. The primary endpoint was the intra-class correlation coefficient (ICC) of the volume of the index tophus measured by US and DECT and its 95% confidence interval (CI 95%).

Inter-reader agreement for assessment of volume using DECT was analysed by the calculation of the intra-class correlation coefficient (ICC) and its CI 95%. Correlation was tested by calculating the Spearman correlation coefficient, as the data were not normally distributed.

Multivariate analysis of the complete data was then performed to search for factors affecting the measurement of the global DECT volume. Multiple linear regression was performed using data on the following nine variables: eGRF, disease duration, BMI, SUA, treatment duration, number of flares over the previous year, current daily alcohol consumption (g/day), diuretic use, and gender. Log transformation of the volume was necessary in order to verify that regression conditions were fulfilled (homoscedasticity and normality).

The small sample size required the application of a variable selection procedure. The step by step, backward method, based on the Akaïke criterion was chosen. Validation and model performance was assessed graphically using the residuals. The significance level was set at 5%.

## Results

### Population

Overall, US examination and DECT scanning were performed within a 2-week interval in 64 patients, and these were analysed. The characteristics of the population are detailed in Table 1.

**Table 1:** Patients’ characteristics

Characteristic	Value in study sample
Number of patients	64
Male	54 (84.4%)
Age (years)	65.4 ± 14.1
Body mass index	28.5 ± 4.1
High blood pressure	34 (53.1%)
Coronary heart disease	12 (18.8%)
Peripheral arterial disease	3 (4.7%)
Stroke	7 (10.9%)
Dyslipidaemia	34 (53.1%)
Diabetes mellitus	17 (26.6%)
Chronic sleep apnoea	9 (14.1%)
Psoriasis	6 (9.4%)
Ongoing diuretic treatment	15 (23.4%)
History of renal stones	11 (17.2%)
Familial history of gout	14 (21.9%)
eGFR (CKD-EPI/MDRD)	75.6 ± 25.6
CKD 3 or worse (clearance <60 ml/min)	16 (25.0%)
Daily alcohol consumption (g)	14.5 ± 22.7
Excessive sweetened beverages	8 (12.5%)
Excessive purine-rich foods	17 (26.6%)
2015 ACR/EULAR diagnosis score	12.8 ± 2.4
Crystal-proven gout	11 (17.2%)
Clinical tophi	21 (32.8%)
Gout duration (years)	12.8 ± 12.3
Number of flares per year	4 ± 6
Ongoing urate-lowering therapy (*n*)	43 (67.2%)
Urate-lowering therapy	
Febuxostat	17 (39.5%)
Allopurinol	24 (55.8%)
Benzbromarone	1 (2.3%)
Probenecid	1 (2.3%)
Serum uric acid level (mg/dl)	7.36 ± 2.55

Quantitative variables are expressed as mean **±** standard deviation, and qualitative variables as number (percentage). *eGFR* estimated glomerular filtration rate, *CKD* chronic kidney disease, *MDRD* modification of diet in renal disease, *ACR* American College of Rheumatology, *EULAR* European League Against Rheumatism

### US and DECT findings

**Fig. 1:**
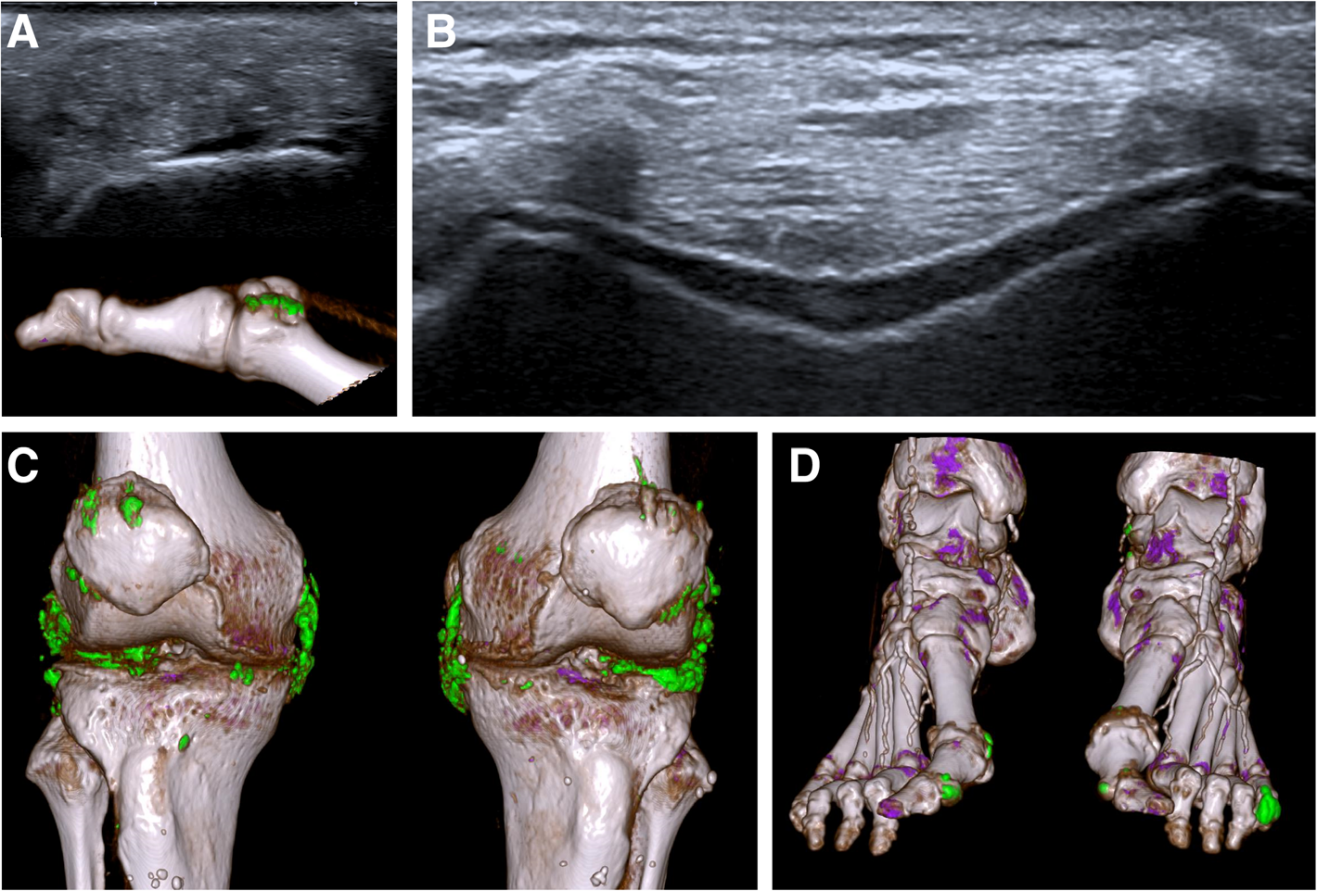
**a** Tophus of the first metatarsophalangeal joint assessed by ultrasonography (US) (*top*) and dual-energy computed tomography (DECT) (*bottom*). **b** US double contour sign. **c** DECT scans of the knees. **d** DECT scans of the feet

Of the 64 patients included, 34 presented with at least one tophus on US examination (Fig. 1a). The largest tophus, defining the index tophus, was mainly localised on the first MTP (Fig. 1b). Almost all patients (95.3%) presented with at least one joint positive for the DC sign (Table 2).

**Table 2:** Ultrasonographic identification of tophi and the double contour (DC) sign

Ultrasonic features	Prevalence	Missing values (*n*)
Detection of at least one US tophus	34 (53.1%)	
Localisation of the largest US tophus		
Right MTP	17 (50%)	
Left MTP	9 (26.5%)	
Right ankle	0 (0%)	
Left ankle	1 (2.9%)	
Right knee	1 (2.9%)	
Left knee	6 (17.6%)	
US tophus volume (cm^3^)	2.5 ± 6.5	
At least one joint with positive DC sign	61 (95.3%)	
Localisation of DC signs		
Right MTP	48 (81.3%)	2
Left MTP	51 (86.4%)	2
Right TC	16 (26.7%)	1
Left TC	18 (30%)	1
Right FP	16 (25%)	
Left FP	14 (21.9%)	

Quantitative variables are expressed as mean ± standard deviation, and qualitative variables as number (percentage). *MTP* metatarsophalangeal joint, *TC* talo-crural joint, FP femoro-patellar joint

The volumes of urate deposition measured by DECT were 5.6 cm^3^ (±17.1) in the feet and 7.3 cm^3^ (±31.1) in the knees, with an average overall deposition of 13.6 cm^3^ (±48.6) (Fig. 1c and d) (Table 3).

**Table 3:** Dual-energy computed tomography (DECT) volume measurements of urate deposits and tophi expressed in cm^3^

DECT features	Total population	Patients with US tophi	Patients without US tophi
	(N = 64)	(N = 34)	(N = 30)
Volume of urate deposition of the knees	7.3 ± 31.1	12.5 ± 40.6	0.4 ± 0.6
Volume of urate deposition of the feet	5.6 ± 17.1	9.7 ± 22.4	0.7 ± 0.6
Volume of overall urate deposition	13.6 ± 48.6	22.6 ± 62.5	1.1 ± 1.2
Volume of the index tophus	N/A	1.3 ± 3.2	N/A
Volume of the largest urate deposit seen by DECT	1.4 ± 5	2.6 ± 6.7	0.1 ± 0.1

Variables are expressed as mean ± standard deviation. *N/A* not applicable

### Inter-reader reliability for DECT volume assessment

Inter-reader agreement for assessment of global urate deposition in the knees and feet was perfect with an ICC of 1 (1 − 1). Inter-reader agreement for the measurement of the index tophus was good with an ICC of 0.69 (0.47–0.83). Inter-reader agreement for the determination of the largest tophus seen on DECT and its measurement was excellent with an ICC of 0.98 (0.97–0.99).

### Agreement between DECT and US for tophus measurement

**Fig. 2:**
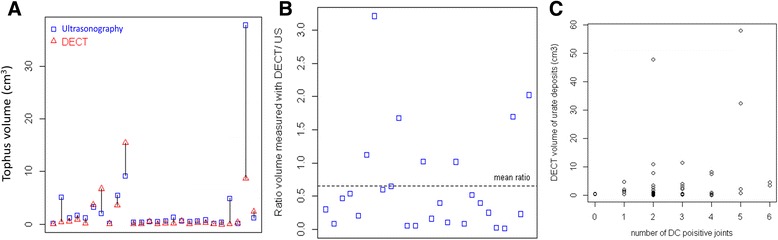
Dual energy computed tomography (*DECT*) and ultrasonography (US) provide different quantification of urate deposition. **a** Comparison of each individual tophus volume as measured by DECT (*triangles*) and US (*squares*). **b** Ratio of the volume measured with DECT and US for each individual tophus. **c** Volume of overall urate deposition measured by DECT according to the number of joints presenting with the US double contour (*DC*) sign

Of the 34 index tophi identified using US, 8 did not exhibit any urate deposition on the DECT scans: all 8 tophi not identified on DECT were localised on the first MTP and had an average US volume of 0.7 cm^3^ (±0.6). The ICC was calculated upon the measurement of the 26 index tophi identified by US and by DECT. The ICC for the measurement of the index tophus was poor with a value of 0.45 (0.1–0.71). When considering only the 19 tophi localised in the feet, the agreement was good, with an ICC of 0.75 (0.45–0.89). Of note, the index tophus was not the largest tophus identified by DECT in 19/34 patients (55.9%). Among the 30 patients without any tophus visualised on US, 7 had tophi identified on DECT: among these tophi, 3 were seen in the popliteal region, 2 in the quadricipital tendon, 1 on the tarsal bones and 1 on the fifth toe. Except for the quadricipital tendon and tarsal bones, the other areas were not intended to be explored by US according to the protocol. The three tophi that were identified by DECT and in the anatomical area examined by US had an average volume of 0.06 cm^3^ (±0.01). The average ratio between index tophi volume as assessed by DECT and US was 0.65 (Fig. 2b).

### Correlation between US features and volume of overall urate deposits assessed by DECT

The number of DC-positive joints did not correlate at all with the volume of overall deposits assessed by DECT, with a very low Spearman correlation coefficient of 0.23. The volume of index tophi assessed by US correlated well with the volume of overall deposits assessed by DECT, with a Spearman correlation coefficient of 0.63, which was, however, lower than obtained for assessment of the largest tophus identified by DECT (*ρ* = 0.75). Furthermore, the presence of tophi on US was marked by far greater volumes of urate deposits using DECT than in their absence, with a volume of 22.6 cm^3^ (±62.5) versus 1.1 cm^3^ (±1.2), respectively.

### Factors correlated with the volume of overall urate deposition assessed by DECT

The automatic procedure in the multivariate model selected three of the nine candidate variables as explicative (Table 4). Greater daily alcohol intake and longer disease duration positively influenced the volume of urate deposition. Inversely, eGFR was negatively associated with urate deposition. Notably, SUA levels were not independently associated with the extent of urate deposition identified by DECT.

**Table 4:** Identification of factors influencing assessment of volumes of overall deposition by dual-energy computed tomography (DECT)

Factors tested in the model	Association between the factor and volume of deposits assessed by DECT	β regression coefficient	*P* value
Glomerular filtration rate	Yes	-0.024	0.008
Gout duration	Yes	0.041	0.03
Body mass index	No		
Number of flares over the last year	No		
Gender	No		
Serum uric acid level	No		
Daily alcohol consumption	Yes	0.02	0.03
Urate lowering therapy duration	No		
Diuretic use	No		

*P* values <0.05 indicate significant association

## Discussion

This cross-sectional study has shown that US and DECT do not provide the same quantitative assessment of urate burden in the feet and knees. Particularly, the two techniques were discordant for assessing tophi volume, with DECT generally underestimating the volume compared to US. The agreement was improved when considering only tophi localised in the feet, yet small tophi of the first MTP often did not demonstrate any deposits on DECT. The number of joints with a positive DC sign did not correlate with the volume of urate deposition in the soft tissues measured by DECT. Of note, the high prevalence of the DC sign despite urate-lowering therapy (ULT) further suggests that ULT clears joints of the DC sign if SUA levels are sufficiently lowered, which was not the case in this cohort [22].

Recurrent underestimates of tophi size by DECT can be explained by the fact that DECT only measures the crystal content of the tophus, whereas US measures the whole volume. Dalbeth et al. have shown that DECT deposits are scattered across the tophus and are surrounded by soft tissue [9], which is consistent with a previous histological analysis of tophi from the same team [23]. US measurements of tophi, like physical examination, provides an assessment of the overall tophaceous structure, soft tissue included [9]. It is probable that DECT provides a more accurate assessment of the crystal deposition itself within the tophus, regardless of the surrounding cellular organisation [24]. The level of accuracy of the real volume of crystals assessed by DECT remains unclear as it has not been thoroughly explored so far [25]. Attempts to compare tophi visualised by DECT to histological findings have been inconclusive, as the methods used to preserve the MSU crystals have an impact on tophus volume [26]. The ratio between the two techniques of 0.65 for assessment of tophi volume could be an approximation of the average crystal content of the tophi.

Difficulties in precisely outlining tophi using US and DECT also account for discrepancies between DECT and US volume assessments. The echogenicity and the size of the posterior acoustic shadow are variable from one tophus to another [27]. In certain cases, combination of hypoechoic tophus with a large posterior acoustic shadow complicates tophus outlining, and gives a more random measurement of its size (Additional file 1). Tophus shape itself can lead to overestimation of tophus volume by US, particularly in cases of first MTP tophi where the tophus often overlies the joint rather than having a spherical formation (Additional file 2). In these cases, US volumes are inevitably overestimated because using the largest measure for each of the three dimensions to calculate the volume is not adapted to such “bean-shaped” tophaceous formations. Some tophi had larger volumes when measured by US than by DECT. There are also difficulties in outlining tophi when they are very large and within tophi aggregates (see Additional file 2: Figure S2b).

US identified tophi that were not identified using DECT, and DECT identified individual deposits that were ot identified using US. Particularly, several tophi on the first MTP were missed by DECT (Additional file 3). A legitimate explanation would be the notion that crystal deposition volume within these generally small tophi is too small to be detected by DECT (0.3 mm resolution threshold), rather than the notion of US misdiagnosis, given the fact that a first MTP tophus is a very common feature identified by US in patients with gout [14, 28]. On average, the volumes of urate deposits visualised using DECT in patients without US tophi were small, further highlighting the known good sensitivity of US for detection of tophi. In addition, this further suggests that tophus formation requires an initial minimal volume of urate deposition [29]. Of note, another reason for DECT demonstrating tophi not shown by US would be due to their intra-osseous localisation, but this was not encountered in this cohort [30].

We acknowledge that this study has some limitations. First, the results might have been different if other joints had been chosen for examination. The analyses were limited to the knees and feet for practical reasons (limiting time-consuming US examination of numerous joints) and for patient safety (reducing patient irradiation using DECT). There has not been a general consensus so far on which joints should be examined to assess urate burden in gout [18]. The knees and feet were selected among other joints for volumetric assessment because they are known to have more prevalent deposits identified by DECT and US [14]. Second, other US features, and particularly hyperechoic aggregates, were not taken into account as signs of urate joint deposition in addition to the consensual DC sign suggested by the Outcome Measures in Rheumatology (OMERACT) Gout Working Group [18]. Since the OMERACT Ultrasound Working Group has clearly shown that the reliability of these signs is poor [27], they were not included in this study. Third, DECT measurements are known to be impaired by artefacts such as nailbeds, movement, or vascular structures, which can lead to an overestimate of automatic overall volume measurements of knees and feet [21]. These artefacts are of concern for diagnostic DECT because single urate spots could be misleading. This cannot be said for quantitative assessment as these artefacts have negligible volumes compared to urate deposits in patients with established gout (particularly when deposits are manually retrieved), as shown by the small overall volumes in patients without tophi. In this study, the box containing the tophus of interest was defined by the operator, so artefacts could be visually excluded and did not interfere with the primary endpoint.

## Conclusion

US and DECT are efficient tools for the assessment of urate deposition, each with advantages and drawbacks. Providing the equipment is available, DECT allows for easy and largely automated acquisition. US on the other hand is largely available but is operator-dependent and its reliability depends on the availability of trained radiologists or rheumatologists. In the prospect of using US and DECT for monitoring the urate burden of treated patients over time, our results imply that the techniques available today cannot provide interchangeable values for repeated measurements of target tophi. It is, however, noteworthy that tophus size assessed by US correlates with assessment of urate deposition by DECT. Optimising the assessment of urate volume, and particularly tophus size, is a prerequisite before considering DECT and US as tools for monitoring urate deposition in the follow up of patients on treatment. Particularly, the question of how to optimise the DECT image processing software is a determining factor, as changing parameters can lead to the appearance of new deposits (and artefacts?) and can significantly modify the measured volumes of urate deposition [31]. In addition, future studies are necessary to determine whether there is correlation between DECT and US in their sensitivity to change.

## Additional files

Additional file 1: Tophus with large posterior acoustic shadow. (TIF 1894 kb)

Additional file 2 Bean-shaped tophus of the first metatarsophalangeal joint seen in (A) ultrasonography, (B) 3D dual-energy computed tomography (DECT) imaging and (C) 2D DECT imaging. For DECT images, urate appears in green and calcium in blue. (TIF 2889 kb)

Additional file 3 Tophus of the first metatarsophalangeal joint (A) detected by ultrasonography but (B) not seen with dual energy computed tomography. (TIF 2781 kb)

## Abbreviations

ACR: American College of Rheumatology
BMI: Body mass index
CKD: Chronic kidney disease
CRP: C-reactive protein
DC: Double contour
DECT: Dual-energy computed tomography
eGFR: Estimated glomerular filtration rate
EULAR: European League Against Rheumatism
ICC: Intra-class correlation coefficient
MSU: Monosodium urate
MTP: Metatarsophalangeal joint
OMERACT: Outcome Measures in Rheumatology
SUA: Serum uric acid
US: Ultrasonography
