# Supplementary figures and images for: Ultrasonography and dual-energy computed tomography provide different quantification of urate burden in gout: results from a cross-sectional study
Source: Arthritis Res Ther. 2017 Jul 21;19:171. doi: 10.1186/s13075-017-1381-2 (PMC5521183; doi:10.1186/s13075-017-1381-2)

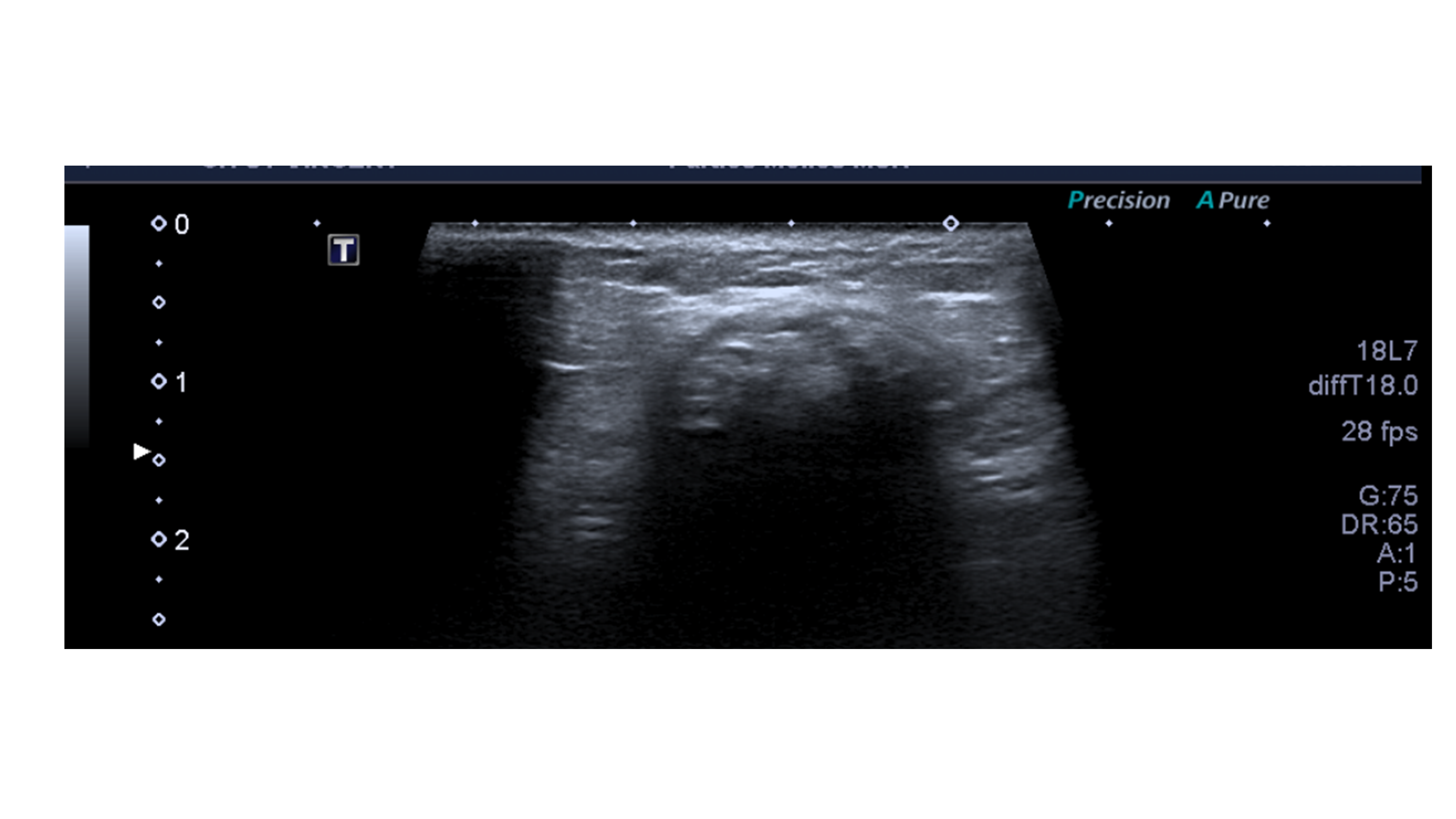

Supplement: Supplementary file 1 — Tophus with large posterior acoustic shadow. (TIF 1894 kb) [file 13075_2017_1381_MOESM1_ESM.tif]

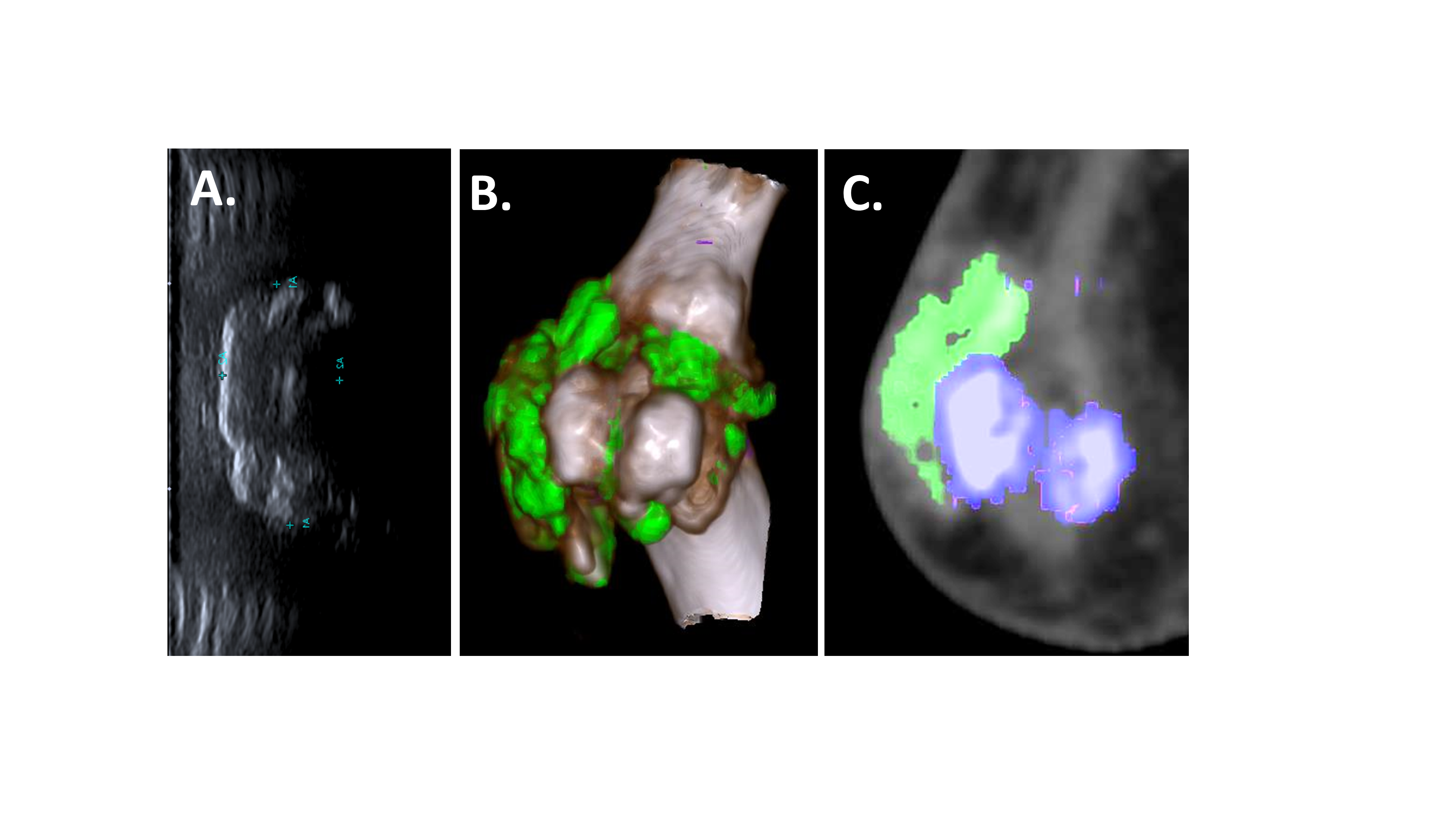

Supplement: Supplementary file 2 — Bean-shaped tophus of the first metatarsophalangeal joint seen in (A) ultrasonography, (B) 3D dual-energy computed tomography (DECT) imaging and (C) 2D DECT imaging. For DECT images, urate appears in green and calcium in blue. (TIF 2889 kb) [file 13075_2017_1381_MOESM2_ESM.tif]

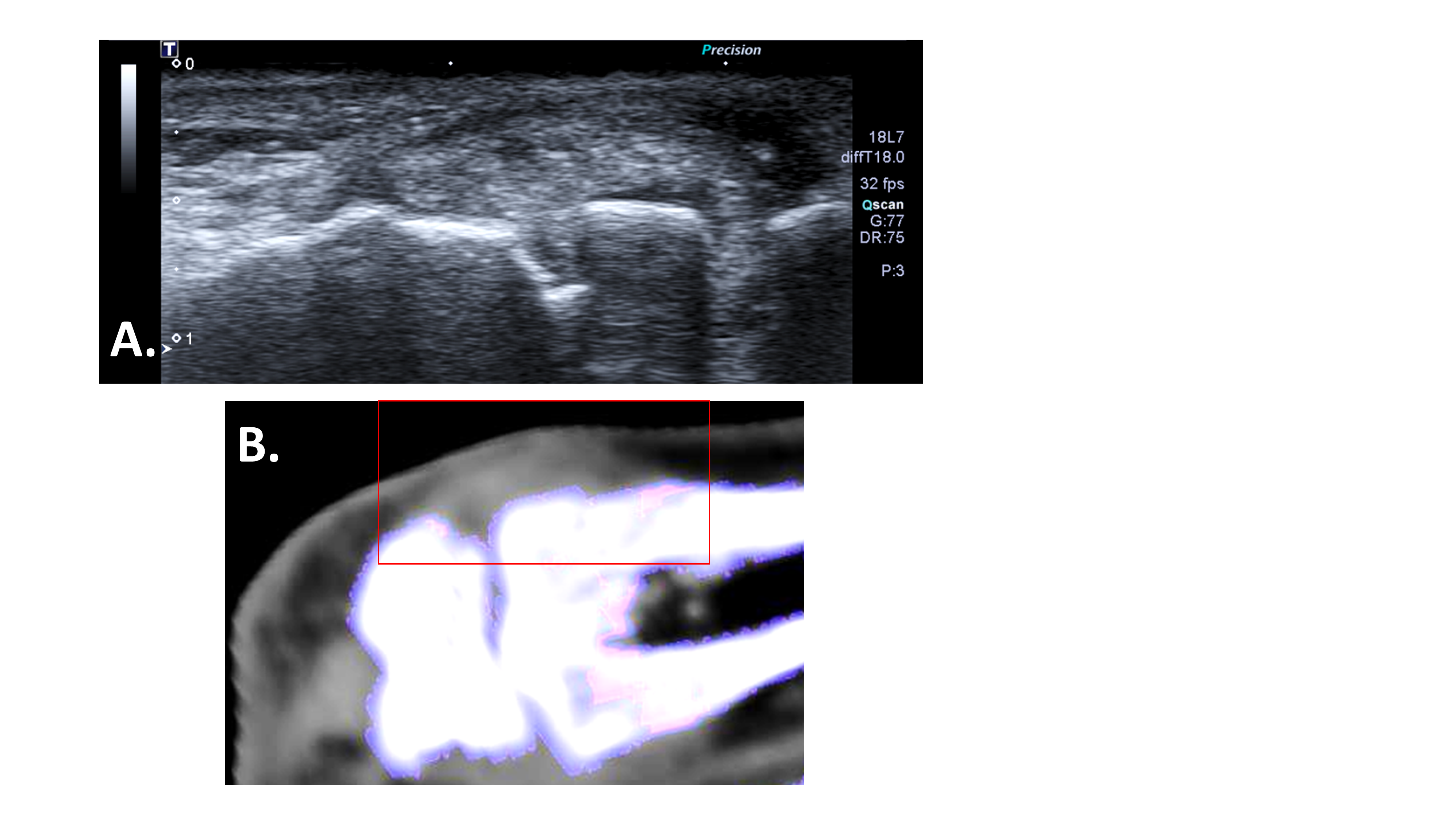

Supplement: Supplementary file 3 — Tophus of the first metatarsophalangeal joint (A) detected by ultrasonography but (B) not seen with dual energy computed tomography. (TIF 2781 kb) [file 13075_2017_1381_MOESM3_ESM.tif]
